# Impact of Body Mass Index on Robotic Surgery Outcomes in Early-Stage Endometrial Cancer: A Retrospective Cohort Study

**DOI:** 10.3390/cancers17213570

**Published:** 2025-11-05

**Authors:** Dimitrios Papageorgiou, Eleftherios Zachariou, Ioakeim Sapantzoglou, Elias Tsakos, Emmanouil M. Xydias, Dimitrios Dimitroulis, Nikolaos Plevris

**Affiliations:** 1 Department of Gynecology, Athens Naval and Veterans Hospital, 11521 Athens, Greece; 2 1st Gynecology Department, Metropolitan General Hospital, 15562 Athens, Greece; 3 1st Department of Obstetrics and Gynecology, National and Kapodistrian University of Athens, 11527 Athens, Greece; 4 Department of Obstetrics and Gynecology, EmbryoClinic IVF, 55133 Thessaloniki, Greece; 5 2nd Department of Surgery, Laiko General Hospital, National and Kapodistrian University of Athens, 11527 Athens, Greece

**Keywords:** endometrial cancer, robotic-assisted surgery, robotic hysterectomy, robotic surgery, sentinel lymph node biopsy, body mass index, obese patients, obesity

## Abstract

**Simple Summary:**

Excess body weight greatly increases the risk of endometrial cancer and at the same time makes surgery harder, because access to the pelvis is more difficult. Robotic surgery is a minimally invasive technique that may overcome these challenges, but data in morbidly obese patients are still limited. We reviewed 54 women with early endometrial cancer who underwent a robotic hysterectomy with bilateral salpingo-oophorectomy, together with lymph nodes sampling. We compared results across body mass index groups, including women with severe obesity. Operating time, hospital stay, complication rates and sentinel lymph nodes detection rates were similar among all groups. These findings support robotic surgery as a safe, effective standard, even for morbidly obese patients.

**Abstract:**

**Background/Objectives**: Obesity is a well-established risk factor for endometrial cancer and presents challenges for surgical management. Robotic-assisted surgery offers a minimally invasive approach with potential benefits for obese patients. This study sought to assess the impact of body mass index (BMI) on surgical performance and short-term outcomes in patients undergoing robotic surgery for early-stage endometrial cancer, focusing on follow-up and perioperative treatment. **Methods**: A retrospective analysis was conducted on 54 patients with early-stage endometrial cancer who underwent a robotic total hysterectomy, bilateral salpingo-oophorectomy, and indocyanine green sentinel lymph node biopsy between January 2021 and December 2024 at two tertiary centers. Patients were stratified by body mass index. Surgical variables, sentinel lymph node detection rates, peri- and postoperative complications, length of hospital stay, and short-term oncologic outcomes were assessed. Statistical comparisons were performed using ANOVA, chi-square tests, and Pearson’s correlation analysis. **Results**: The mean patient age was 59.7 years, with a mean BMI of 31.1 kg/m^2^. Bilateral sentinel lymph node detection was successful in 87% of cases, with no significant differences between BMI groups. Console time, hospital stay, and complication rates were comparable across BMI categories. Console time positively correlated with the number of lymph nodes removed (r = 0.302, *p* = 0.026), but not with BMI. At a mean follow-up of 24.4 months, no recurrences were observed. **Conclusions**: Robotic surgery for early-stage endometrial cancer is safe and effective regardless of BMI, including in patients with Class III obesity. BMI does not negatively impact surgical or short-term oncologic outcomes, supporting robotic surgery as an optimal approach in obese endometrial cancer patients.

## 1. Introduction

Endometrial Cancer (EC) represents the leading gynecologic malignancy in developed countries. According to United States cancer statistics, in 2025, 69,120 new uterine cancer diagnoses will take place, while deaths from EC are estimated to reach 13,860 [1]. The occurrence of EC has been steadily increasing and has approximately doubled over the last 20 years. The most common histological subtypes consist of endometrioid and uterine serous carcinoma (USC), with the latter being more aggressive. USC accounts for about 10% to 15% of the EC cases; nevertheless, it causes up to 40% of all EC deaths. More often, it is diagnosed at a more advanced stage, exhibits high-grade histology, and is accompanied by a poor prognosis [2,3]. The initial appearance of abnormal uterine bleeding allows patients to obtain prompt clinical care which results in successful early diagnosis [4].

The majority (75–80%) of endometrial cancer cases at diagnosis present with early-stage disease (FIGO stage I-II) [5]. The prognosis for these cases remains favorable because stage I tumor patients achieve five-year survival rates above 90% [1]. Obesity represents a significant modifiable risk factor for endometrial cancer (EC), with a nearly 60% increase in disease risk associated with each 5 kg/m^2^ increment in Body Mass Index (BMI) [6]. The rising prevalence of obesity presents significant challenges for surgeons in delivering effective surgical management for this demographic.

Over the past years, a notable progression in the diagnosis and treatment of endometrial malignancies has been the establishment of molecular classification systems, with the Cancer Genome Atlas offering an extensive classification, delineating endometrial cancer into four distinct genomic categories: POLE ultramutated (POLEmut), microsatellite instability hypermutated or mismatch repair deficiency (MSI-H or MMRd), p53 abnormal (p53abn), and non-specific molecular profiler (NSMP) [7]. The recent FIGO 2023 endometrial cancer staging system has incorporated this molecular categorization, representing a significant advancement in the stratification of endometrial cancer risks beyond the established conventional histologic assessments, offering the clinicians a helpful tool in the pursuit of treatment alternatives [8,9].

The standard treatment for early-stage endometrial cancer (EEC) requires a total hysterectomy with bilateral salpingo-oophorectomy (TH-BSO), while performing lymph node assessment through either pelvic and para-aortic lymphadenectomy or sentinel lymph node biopsy (SLNB), depending on institutional protocols and tumor characteristics [7]. Minimally invasive techniques have predominantly supplanted laparotomy for early-stage endometrial cancer, owing to lower morbidity and comparable oncologic results. Consequently, laparoscopy is now advised as the preferred option for suitable patients [8]. Robotic-assisted surgery provides significant ergonomic and visual benefits, especially for obese patients where exposure and dexterity may be limited [10]. Numerous studies indicate that robotic surgery decreases blood loss, length of hospital stay, and complications; however, the impact of increasing BMI on perioperative and short-term oncologic outcomes is still unclear [11,12,13]. Evidence is notably limited for morbidly obese patients (BMI ≥ 40 kg/m^2^), who constitute the population with the highest surgical risk.

This study investigates the impact of BMI on surgical performance, complication rates, and short-term oncologic outcomes in patients undergoing robotic surgery for early-stage endometrial cancer, with an emphasis on morbidly obese patients, a subgroup with limited published evidence, to gather real-world data for evidence-based surgical decisions.

## 2. Materials and Methods

We conducted a retrospective cohort study at two Greek tertiary centers, including Metropolitan General Hospital in Athens and EmbryoClinic in Thessaloniki. The objective was to investigate the influence of BMI on both surgical and oncologic outcomes in patients undergoing robotic-assisted surgery for early-stage endometrial cancer.

Patients diagnosed with early-stage endometrial carcinoma, preoperatively, who underwent robotic-assisted TH-BSO and bilateral SLNB using ICG fluorescence mapping between January 2021 and December 2024 were included. Patients with preoperatively advanced-stage disease (FIGO Stage III or IV) and patients with incomplete/missing perioperative or pathological data, as well as patients who underwent open or laparoscopic surgery, have been excluded from our research.

MRI served as the principal modality for staging in preoperative imaging. Transvaginal ultrasound (TVUS) was utilized when MRI was contraindicated or technically limited. Standardization was applied to postoperative follow-up and adjuvant therapy management. In the initial month following surgery, each patient received a physical examination conducted by the operating surgeon to evaluate recovery and identify early complications. Upon the availability of the final histopathology results, the institutional oncology council reviewed all cases and assessed the necessity for adjuvant treatment in alignment with ESGO/ESMO–ESTRO guidelines [4]. Patients in need of adjuvant therapy were directed to the medical oncology clinic, where treatment and follow-up surveillance were organized. Follow-up typically involved a physical examination every 3 to 6 months during the initial three years, every 6 to 12 months for the subsequent three years, and annually thereafter. Imaging studies, such as pelvic ultrasound or MRI, were conducted when clinically indicated or when recurrence was suspected.

BMI was categorized according to the World Health Organization (WHO) classification into underweight (<18.5 kg/m^2^), normal weight (18.5–24.9 kg/m^2^), overweight (25.0–29.9 kg/m^2^), Class I obesity (30.0–34.9 kg/m^2^), Class II obesity (35.0–39.9 kg/m^2^), and Class III obesity (≥40.0 kg/m^2^).

All surgeries were performed using the da Vinci^®^ robotic surgical platform (Intuitive Surgical, Sunnyvale, CA, USA), by two experienced gynecologic oncologists, who perform more than 50 robotic cases yearly. Sentinel lymph node mapping was performed through cervical injection of ICG at a concentration of 1.25 mg/mL, achieved by dissolving 25 mg of ICG in 20 mL of sterile water. A total of 4 mL (5 mg) was administered intracervically at the 3 o’clock and 9 o’clock positions: 1 mL was delivered submucosally and 1 mL was injected deeply (1 cm) at each site immediately prior to trocar placement. Intraoperative visualization was achieved using near-infrared fluorescence imaging. A standard robotic-assisted hysterectomy and a bilateral salpingo-oophorectomy were then performed. In cases where bilateral SLN mapping was unsuccessful, side-specific lymphadenectomy was conducted according to institutional protocols. A pelvic lymphadenectomy was conducted when bilateral sentinel lymph node mapping failed, or in instances of Grade 3 or non-endometrioid histology, deep myometrial invasion exceeding 50%, or the presence of suspicious or enlarged lymph nodes observed on preoperative MRI. The indications adhered to institutional protocols consistent with the most recent ESGO/ESTRO/ESP guidelines for managing patients with endometrial carcinoma [7].

Data collected included patient demographics (age, BMI, American Society of Anesthesiologists (ASA) physical status), surgical variables (console time, estimated blood loss, intraoperative and postoperative complications, length of hospital stay, reoperation, readmission, and conversion rates), and oncologic variables (tumor grade, lymphovascular space invasion (LVSI), and SLN detection success). Perioperative and oncologic data were collected from institutional electronic medical records.

Continuous variables were expressed as means ± standard deviation (SD) and ranges, while categorical variables were presented as frequencies and percentages. Differences in continuous variables among BMI categories were assessed using one-way analysis of variance (ANOVA), and categorical variables were compared using chi-square tests. Pearson’s correlation coefficient was used to evaluate associations between BMI, console time, and length of stay (LOS). A *p*-value of less than 0.05 was considered statistically significant. All statistical analyses were conducted using IBM SPSS Statistics for Windows, Version 29.0 (IBM Corp., Armonk, NY, USA).

The study was approved by the Institutional Review Board of the Metropolitan General Hospital (Approval Number: 97/27.06.25). Due to the retrospective design, informed consent requirements were waived.

## 3. Results

In total, 54 patients were included in our study. The mean age of the patients included was 59.7 years (range: 38–84 years, SD 11.3), with a mean BMI of 31.1 kg/m^2^ (range: 19.1–53.7 kg/m^2^, SD 9.4). According to the WHO BMI classification, 19 patients (35.2%) had normal weight, 10 (18.5%) were overweight, and 25 (46.3%) were obese, subdivided into Class I (*n* = 11, 20.4%), Class II (*n* = 2, 3.7%), and Class III (*n* = 12, 22.2%), indicating that the majority of the patients (35 out of 54) fell into the overweight or obese categories (Table 1).

The mean ASA physical status score was 1.83, while the majority of the patients (*n* = 42, 77.8%) were postmenopausal. Most patients did not report smoking, type II diabetes, or vascular disease on their medical history. However, 25 out of 54 (46.3%) suffered from dyslipidemia, while the majority of them were overweight or obese. Additionally, 39 out of 54 patients (72.2%), reported a history of abdominal surgery. Higher BMI categories demonstrated an increased prevalence of adverse clinical and surgical risk factors. Specifically, Class III obese patients (ΒΜΙ ≥ 40.0 kg/m^2^) had the highest rates of diabetes mellitus type II (66.7%), ASA III classification (66.7%), dyslipidemia (58.3%), and prior abdominal surgeries (100%). Patient characteristics stratified by BMI are shown in Table 1.

The mean robotic console operative time was 121.4 min (range: 52–250 min, SD 46.7). In 25 out of 54 cases (46.3%), console time exceeded 120 min. Prolonged console time (≥120 min) was more frequently observed in obese Class I patients (63.6%), followed by overweight patients (50%), obese Class II patients (50%), obese Class III patients (41.7%), and normal-weight patients (36.8%) (Table 2). One-way ANOVA analysis showed no significant difference in console operative time among the BMI groups (F(4,49) = 0.236, *p* = 0.917). Pearson’s correlation analysis did not report a statistically significant correlation between BMI and console time (r = 0.019, *p* = 0.890). A chi-square test showed no association between BMI and console time categorized as <120 min vs. ≥120 min (χ^2^ = 2.183, *p* = 0.702) (Supplementary File S1).

**Figure 1:**
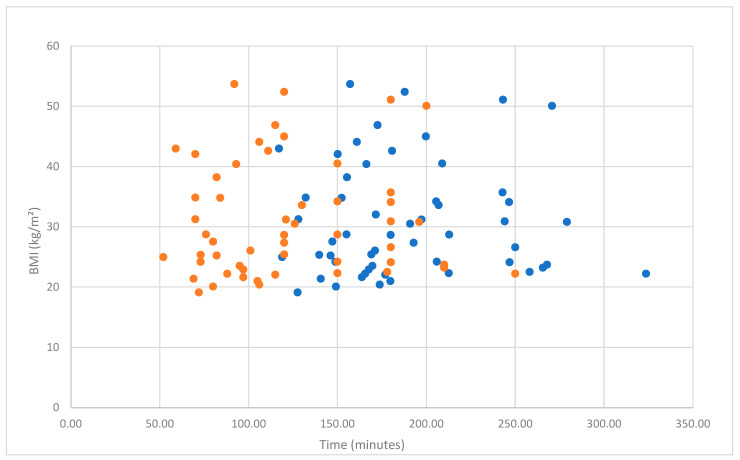
Scatterplot of the relationship between BMI and total operative time (blue dots) and console time (orange dots).

**Figure 2:**
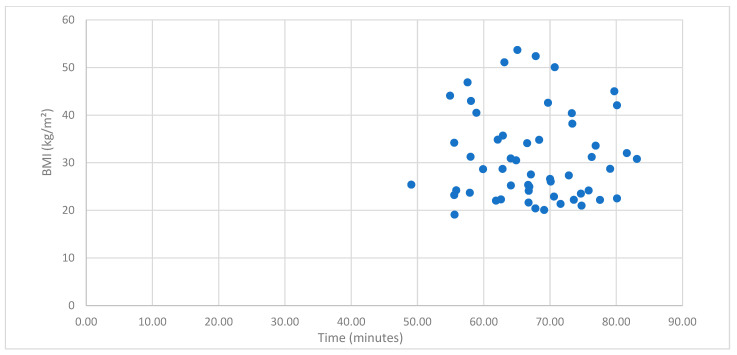
Scatterplot of the relationship between BMI and non-console time (docking + anesthesia + patient transit).

Regarding subgroup analysis, mean robotic console operative time in morbidly obese patients (BMI ≥ 40) was 118 min, while in non-morbidly obese patients (BMI < 40) it was 122.33 min. A t-test revealed that, although the mean robotic console operative time was slightly higher among non-morbidly obese patients, there is no statistically important difference between these two groups (t(52) = 0.281, *p* = 0.780). The mean total operative time in morbidly obese patients (BMI ≥ 40) was 184.58 min, while in non-morbidly obese patients (BMI < 40) it was 189.97 min. Subsequently, the t-test revealed that there is no statistically important difference in mean total operative time between patients of the two groups (t(52) = 0.350, *p* = 0.728). Mean non-console time (docking + anesthesia + patient transit time) among morbidly obese patients was 66.59 min, while among non-morbidly obese patients it was 67.64 min. Similarly, the t-test revealed no statistically significant difference in non-console time between groups (t(52) = 0.397, *p* = 0.693). Our results indicate that ΒΜΙ ≥ 40 does not increase surgical times in patients who undergo robotic-assisted surgery for early-stage endometrial cancer. (Supplementary File S1). Comparable operative times among different BMI groups are demonstrated in Figure 1 and Figure 2 and Table 2 and Table 3.

**Figure 3:**
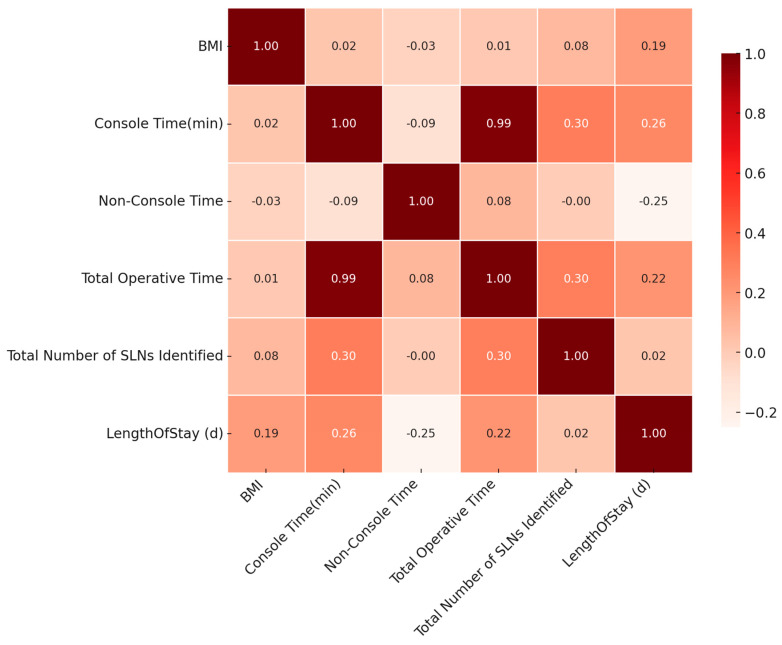
Correlation matrix heatmap shows the relationship between BMI, console time, non-console time, total operative time, total number of SLNs extracted, and length of stay.

Sentinel lymph node detection was successful bilaterally in 87.0% of patients (47/54), with unilateral detection in five cases (9.3%). SLN detection was unsuccessful in only two cases (one patient with normal BMI and one patient of obese Class I category). Bilateral mapping was achieved most frequently among patients of obese Class III category (11 out of 12, 91.7%), followed by overweight patients (9 out of 10, 90%). Regarding SLN anatomical location, external iliac and obturator regions were most commonly sampled, with only one SLN identified in the presacral space (Table 2). The rate of successful SLN detection, whether bilateral or unilateral, was uniformly high across all BMI categories, with no statistically significant association observed between BMI classification and SLN mapping status (χ^2^ = 6.208, *p* = 0.624). Additionally, SLN anatomical location (external iliac, obturator, or pre-sacral) did not differ significantly among BMI groups (χ^2^ = 8.821, *p* = 0.358), supporting the technical feasibility of nodal retrieval even in obese patients. The only statistically significant correlation observed was between console time and the number of lymph nodes extracted (r = 0.302, *p* = 0.026). BMI, on the other hand, did not correlate with the number of SLNs extracted (Figure 3). The mean number of extracted SLNs according to final pathology in morbidly obese patients was 5.25, while in non-morbidly obese patients it was 5. T-test analysis suggests that the difference in SLNs identified is not statistically significant (t(52) = −0.166, *p*= 0.869) (Supplementary File S1).

**Figure 4:**
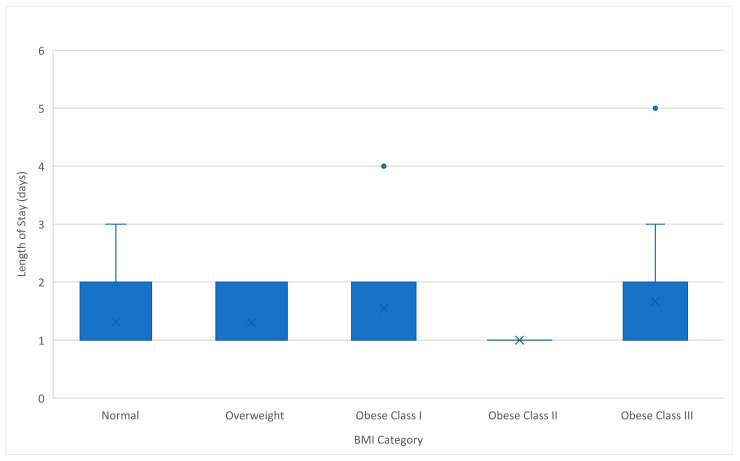
Boxplot of the relationship between length of stay and different BMI categories.

Postoperative outcomes were similarly favorable across BMI categories. The mean postoperative length of hospital stay (LOS) was 1.43 days (range: 1–5 days, SD 0.81). A prolonged hospital stay (>1 day) was noted in 16 out of 54 patients (29.6%). Subgroup analysis revealed a maximum mean LOS of 1.67 days at obese Class III group followed by obese Class I (1.55 days), normal weight (1.32 days), overweight (1.30 days), and obese Class II group (1 day) (Table 2 and Figure 4). The LOS was not significantly different between BMI groups (F(4,49) = 0.585, *p* = 0.675). According to Pearson’s correlation analysis, no significant correlation was observed between BMI and LOS (r = 0.188, *p* = 0.174) (Figure 3). A chi-squared test confirmed that there is no statistically significant correlation between BMI and postoperative length of stay (LOS), even when dichotomized as ≤1 day vs. >1 day (χ^2^ = 1.261, *p* = 0.868). Regarding subgroup analysis, mean LOS in morbidly obese patients was 1.67 days, while in non-morbidly obese patients it was 1.36 days (Table 3). A t-test revealed that, although the mean robotic console operative time was slightly higher among morbidly obese patients, there is no statistically important difference between the two groups (t(52) = −1.164, *p* = 0.250). Additionally, the confidence interval is wide, and the effect size is small, suggesting no clear evidence of prolonging LOS due to morbid obesity. (Supplementary File S1).

Only two patients (3.7%) experienced postoperative complications. Importantly, there were no Clavien–Dindo grade III complications or higher. Additionally, no conversions to laparotomy, no ICU admissions, and no reoperations were recorded. One death was recorded in a 61-year-old postmenopausal patient, 2 months after hospital discharge, due to myocardial infarction. The patient had normal weight (BMI 21.1 kg/m^2^) and a medical history of vascular disease and dyslipidemia (Table 1). Our study did not reveal any patterns indicating greater complication or mortality rates in overweight or obese patient groups.

Regarding oncologic outcomes, the majority of patients had early-stage disease based on FIGO 2023 criteria: IA2 (*n* = 28), IB (*n* = 12), IA1 (*n* = 6), with fewer patients at advanced stages—IIIC1i (*n* = 4), IIB (*n* = 2), IIIA2 (*n* = 1), IIA (*n* = 1). Tumor grade analysis revealed 45 patients (83.3%) with Grade 1 tumors, 5 (9.3%) with Grade 2, and 4 (7.4%) with Grade 3 (Table 4). Histological upgrade between preoperative biopsy and final pathology was observed in six patients (11.1%). These upgrades included changes in tumor grade or the presence of LVSI, which were not evident in initial sampling. The mean follow-up period was 24.4 months (range: 1–67 months, SD 18.1). At the time of the last follow-up, there were no disease recurrences recorded.

## 4. Discussion

### 4.1. Principal Findings

Our cohort of early-stage endometrial cancer patients who underwent robotic-assisted surgery demonstrated non-significant differences among the different BMI groups in terms of console time, detection rates of SLN, perioperative complications, postoperative length of stay, and postoperative morbidity and mortality. Patients with Class III obesity (BMI ≥ 40 kg/m^2^) exhibited operative times, complication rates, and oncologic outcomes similar to those of patients with a lower BMI. Our findings suggest a positive correlation between surgical time and the number of SLNs removed but BMI did not have an impact on this association.

The findings indicate that robotic technology has the potential to alleviate several technical limitations associated with obesity, specifically restricted pelvic visualization and reduced surgeon dexterity, by enhancing ergonomics and precision. The absence of significant performance degradation at elevated BMI levels highlights robotic surgery’s capacity to standardize outcomes across various body types.

### 4.2. Comparison to Existing Literature

In this study, MRI served as the primary method for the preoperative evaluation of myometrial and cervical invasion, while TVUS was utilized in cases where MRI was contraindicated or technically restricted. This protocol aligns with current European recommendations, endorsing MRI as the reference standard for local staging, while acknowledging TVUS as a reliable and accessible alternative with comparable diagnostic accuracy when performed by experienced practitioners. Recent evidence indicates that transvaginal ultrasound (TVUS) can match magnetic resonance imaging (MRI) in assessing the depth of myometrial invasion and cervical involvement, even among obese women where MRI quality may be compromised [14,15,16]. Integrating these modalities guarantees optimal accuracy in preoperative staging while ensuring feasibility and cost-effectiveness across all BMI categories.

Robotic-assisted surgery was introduced in 2005 for the treatment of gynecological conditions [17], demonstrating similar or even superior intraoperative and postoperative outcomes compared to the laparoscopic and open approaches [18,19]. In terms of early-stage endometrial cancer, several studies have demonstrated the reduced risk of surgical morbidity when robotic-assisted surgical staging is performed [20,21,22], findings that were further confirmed by a recent nationwide implementation study which included more than 5500 patients [23].

Obesity is a well-established risk factor for the subsequent development of uterine cancer, with its presence further complicating the treatment approach of those patients. Obesity has been associated not only with surgical complications, but also with anesthesia induction difficulties and postoperative morbidity, given the underlying cardiovascular, respiratory, and metabolic derangements [24,25,26]. As such, the investigation of peri- and postoperative surgical outcomes in obese women treated for early-stage endometrial cancer by robotic-assisted surgical means by several study groups, including ours, seems a reasonable association.

Our study confirmed the results of previous groups with non-significant differences among the different BMI groups regarding operation time, detection rates of SLN, postoperative length of stay, or postoperative morbidity and mortality [27,28]. To be more precise, in a large retrospective cohort (*n* = 1329), a similar rate of postop complications and conversion to laparotomy were reported among the different BMI groups investigated (<30.0, 30.0–39.9, and ≥40.0), as well as a similar length of hospital stay. The importance of these results is further underlined by the fact that the ASA scores were, as expected, different among the BMI groups, which we found to be aligned with our results [27]. The safety of robotic-assisted surgery was further demonstrated by the sample groups ranging from small to nationwide cohorts, revealing that BMI does not seem to influence the peri- and postop results [23,28].

Similarly to other studies, the Clavien–Dindo classification system was used to assess potential surgical complications [29]. In our cohort, only two patients experienced postoperative complications and there were no Clavien–Dindo grade III patients or higher. One patient of the normal weight group, with a history of prior abdominal surgery and dyslipidemia, was readmitted to the hospital 10 days after discharge, due to a fever and abdominal pain. A peritoneal abscess was diagnosed and managed conservatively, via intravenous antibiotics administration (Clavien–Dindo grade II complication). The second patient, classified as Class III obese (BMI 50.1 kg/m^2^), had a background of type II diabetes, vascular disease, dyslipidemia, and previous abdominal surgeries. The patient developed a transient small bowel ileus 24 h after discharge. The ileus resolved by itself after the implementation of supportive measures, such as bowel rest and IV fluids administration (Clavien–Dindo grade I complication). We did not manage to demonstrate an increased complications rate in overweight or obese patients, a finding that is aligned with other authors [30,31,32]. However, Wright et al. revealed an increased risk of medical complications, in particular, bacteremia, respiratory failure, and renal damage, in obese patients treated with an open hysterectomy [33], whereas robotic series, including ours, report complication rates below 5% [28,32]. The results indicate that robotic-assisted surgery significantly lowers obesity-related postoperative risks in comparison to open and traditional laparoscopic methods. The robotic platform’s minimally invasive characteristics likely reduce abdominal wall trauma, enhance ventilation dynamics, and shorten recovery times, thereby alleviating the significant perioperative burden historically seen in obese patients.

In our cohort, BMI did not seem to influence sentinel lymph node detection and sampling, and, as expected, a longer surgery time is associated with a higher number of removed nodes. These results are in accordance with a recent network meta-analysis that showed no difference in terms of complete surgical staging between minimally invasive surgery and laparotomy, with the robotic approach potentially being superior to the laparoscopic one [34]. The highest bilateral detection rate was observed in Class III obese patients, at 91.7%. This unexpected finding may indicate a combination of small-sample statistical variation and the influence of accumulated surgical experience, often referred to as the learning-curve effect. Enhanced fluorescence imaging may compensate for increased adiposity, facilitating successful sentinel lymph node detection in morbidly obese patients. The consistent mapping success across BMI groups demonstrates that robotic surgery can provide oncologically adequate staging irrespective of patient size, thereby challenging the belief that obesity undermines surgical completeness.

According to our results, BMI did not seem to have an impact on the length of stay, and those data are in accordance with the results of other study groups [28]. To be more precise, King et al., in their cohort of 391 patients, did not manage to demonstrate any statistically significant differences among the different BMI groups with their results, which, in fact, were quite similar to ours. The same results were demonstrated by Gracia et al., with no differences among the different BMI groups treated with robotic surgery, though revealing a superiority in terms of length stay of robotic surgery over standard laparoscopy in obese patients [35]. The same results were noted by a recent cohort of 99 patients, in which the mean length of stay was 1.65 ± 0.65 days, with no significant differences when the cohort was subgrouped according to BMI (<30 kg/m^2^ and ≥30 kg/m^2^) [36]. As such, it seems evident from recent studies that BMI does not pose an adverse role in the duration of stay after the robotic management of endometrial cancer.

In view of the robotic console operative time, the literature has produced contradictory results. The mean console time in our cohort (121.4 min (range: 52–250 min, SD 46.7)) seems to be aligned with some studies [37], while it appears shorter than other published reports [20,27,30,37]. This could be explained by the fact that the operations in our cohort were fully performed by highly experienced consultants who were trained in robotic gynecologic surgery. The longer operative times reported by older studies could be due to the only recent implementation of robotic surgery in the treatment of early-stage endometrial cancer [20,38], and, as such, the subsequent lack of proficiency at performing those operations. This study indicates that a BMI of 40 or greater does not extend the total duration of cases, contrasting with previous laparoscopic or open series studies that frequently noted increased operative times associated with higher BMI, attributed to challenges in exposure and trocar placement [8,9,19,30]. The data suggest that robotic articulation and stable camera control mitigate these challenges. The independence of console time from BMI differs from previous laparoscopic findings, suggesting that the robotic platform standardizes operative efficiency across various patient body types. This indicates a significant change in the surgical feasibility for high-BMI patients, highlighting that institutional experience and technology may now be the main factors influencing operative duration, rather than patient size.

It should be highlighted that, although robotic surgery is exhibiting a shorter learning curve compared to laparoscopy, numerous studies suggest that around 50 robotic hysterectomies are necessary to achieve competency and performing at least 30 operations per year seems to be required to maintain such an expertise [39,40].

### 4.3. Limitations

A few limitations of our study need to be acknowledged. The retrospective design of our study seems to be a factor that could potentially introduce bias. Furthermore, the fact that our study sample is limited and the operations were performed by highly experienced and trained gynecologic surgeons poses a risk as to the extent to which our results might be generalized. Adjusted analyses were conducted to mitigate bias; nonetheless, we cannot exclude the possibility that other factors may have affected the results. It should also be underlined that the mean follow-up of 24.4 months of the patients included increases the risk of underreporting late recurrences, potentially affecting our oncological results.

Molecular profiling of endometrial carcinoma, encompassing POLE mutation status, mismatch repair deficiency, p53 abnormality, and the absence of a specific molecular profile, were not routinely accessible for the patients in this cohort. This testing was only recently integrated into clinical practice in accordance with the updated ESGO/ESTRO/ESP guidelines. The lack of molecular classification data constitutes a limitation, given that molecular subtypes have established prognostic and therapeutic significance in endometrial cancer [7]. This limitation does not compromise the validity of the presented surgical outcome analysis.

## 5. Conclusions

Robotic-assisted surgical treatment for early-stage endometrial cancer seems to be a safe, effective, and oncologically acceptable approach. Obesity, which frequently complicates the appropriate management of these patients, does not seem to have an impact on the intraoperative or postoperative results of this method. However, as this approach is a recently established treatment modality, future, larger, and better-designed studies are needed for concrete conclusions to be extracted.

## Figures and Tables

**Table 1:** Patients’ demographics and medical history by BMI category.

BMI Category		Normal (*n* = 19)	Overweight (*n* = 10)	Obese Class I (*n* = 11)	Obese Class II (*n* = 2)	Obese Class III (*n* = 12)
Age Group	30–39	2 (10.5%)	2 (20%)	0	1 (50%)	0
	40–49	9 (47.4%)	4 (40%)	3 (27.3%)	0	1 (8.3%)
	50–59	6 (31.5%)	2 (20%)	2 (18.2%)	0	5 (41.7%)
	60–69	1 (5.3%)	0	5 (45.4%)	1 (50%)	5 (41.7%)
	70–85	1 (5.3%)	2 (20%)	1 (9.1%)	0	1 (8.3%)
Age Mean ± SD (years)		57.8 ± 9.6	58.3 ± 8.2	61.1 ± 7.9	63.5 ± 5.0	60.9 ± 10.2
Menopausal Status	Postmenopausal	14 (73.7%)	6 (60%)	10 (90.9%)	1 (50%)	11 (91.7%)
	Premenopausal	5 (26.3%)	4 (40%)	1 (9.1%)	1 (50%)	1 (8.3%)
ASA Classification	1	9 (47.4%)	5 (50%)	5 (45.4%)	2 (100%)	0
	2	10 (52.6%)	4 (40%)	3 (27.3%)	0	4 (33.3%)
	3	0	1 (10%)	3 (27.3%)	0	8 (66.7%)
Smoking	No	15 (79%)	6 (60%)	11 (100%)	1 (50%)	12 (100%)
	Yes	4 (21%)	4 (40%)	0	1 (50%)	0
Diabetes Type II	No	17 (89.5%)	8 (80%)	9 (81.8%)	1 (50%)	4 (33.3%)
	Yes	2 (10.5%)	2 (20%)	2 (18.2%)	1 (50%)	8 (8.3%)
Vascular Disease	No	18 (94.7%)	10 (100%)	10 (90.9%)	2 (100%)	10 (83.3%)
	Yes	1 (5.3%)	0	1 (9.1%)	0	2 (16.7%)
Dyslipidemia	No	13 (68.5%)	6 (60%)	5 (45.4%)	0	5 (41.7%)
	Yes	6 (31.5%)	4 (40%)	6 (54.6%)	2 (100%)	7 (58.3%)
Abdominal Surgery History	No	8 (42.1%)	4 (40%)	3 (27.3%)	0	0
	Yes	11 (57.9%)	6 (60%)	8 (72.7%)	2 (100%)	12 (100%)

Abbreviations: BMI: body mass index, ASA: American Society of Anesthesiologists, SD: standard deviation. Values are expressed as *n* (%) unless otherwise stated.

**Table 2:** Surgical and perioperative outcomes by BMI category.

BMI Category		Normal (*n* = 19)	Overweight (*n* = 10)	Obese Class I (*n* = 11)	Obese Class II (*n* = 2)	Obese Class III (*n* = 12)
Total Operative Time Mean ± SD (minutes)		192.8 ± 52.4	176.4 ± 44.9	195.9 ± 53.8	199.1 ± 47.2	184.6 ± 45.5
Console Time Mean ± SD (minutes)		125.1 ± 45.3	110.2 ± 41.6	127.0 ± 49.1	131.0 ± 32.5	118.0 ± 40.2
Non-Console Time (Docking + Anesthesia + Patient Transit) Mean ± SD (minutes)		67.7 ± 15.3	66.2 ± 14.8	68.9 ± 15.6	68.1 ± 13.9	66.6 ± 14.8
Console Time	Long ≥ 120 min	7 (36.8%)	5 (50%)	7 (63.3%)	1 (50%)	5 (41.7%)
	Short < 120 min	12 (63.2%)	5 (50%)	4 (36.7%)	1 (50%)	7 (58.3%)
Length of Stay Mean ± SD (days)		1.32 ± 0.5	1.30 ± 0.4	1.55 ± 0.7	1 ± 0.0	1.67 ± 0.9
Length of Stay	Long > 1 day	5 (26.3%)	3 (30%)	4 (36.4%)	0	4 (33.3%)
	Short ≤1 day	14 (73.7%)	7 (70%)	7 (63.6%)	2 (100%)	8 (66.7%)
SLN Detection	Bilateral	17 (89.4%)	9 (90%)	9 (81.8%)	1 (50%)	11 (91.7%)
	Unilateral	1 (5.3%)	1 (10%)	1 (9.1%)	1 (50%)	1 (8.3%)
	None	1 (5.3%)	0	1 (9.1%)	0	0
SLN Location	External Iliac	11 (57.9%)	8 (80%)	8 (72.7%)	0	10 (83.3%)
	Obturator	7 (36.8%)	2 (20%)	3 (27.3%)	2 (100%)	2 (16.7%)
	Presacral	1 (5.3%)	0	0	0	0
Intraoperative Complications		0	0	0	0	0
Perioperative Complications (Clavien–Dindo Grade)		1 (II)	0	0	0	1 (I)

Abbreviations: BMI: body mass index, SD: standard deviation, SLN: sentinel lymph node. Values are expressed as *n* (%) unless otherwise stated.

**Table 3:** Comparison of mean values between morbidly obese (BMI ≥ 40) and non-morbidly obese (BMI < 40) patients.

	BMI Category (kg/m^2^)		*p*-Value
	BMI < 40	BMI ≥ 40	
Mean Console Time (minutes)	122.33 ± 46.7	118 ± 40.2	0.780
Mean Non-Console Time (Docking + Anesthesia + Patient Transit Time) (minutes)	67.64 ± 15.3	66.59 ± 14.8	0.693
Mean Total Operative Time (minutes)	189.97 ± 52.6	184.58 ± 45.5	0.728
Mean Length of Stay (days)	1.36 ± 0.6	1.67 ± 0.9	0.250
Mean Number of Extracted SLNs (SLNs in Final Pathology)	5 ± 2.1	5.25 ± 1.9	0.869

Abbreviations: BMI: body mass index, kg/m^2^: kilograms per meter squared, SLN: sentinel lymph node.

**Table 4:** Oncologic outcomes by BMI category.

BMI Category		Normal (*n* = 19)	Overweight (*n* = 10)	Obese Class I (*n* = 11)	Obese Class II (*n* = 2)	Obese Class III (*n* = 12)
Endometrial Cancer Type	Endometrioid	18 (94.7%)	10 (100%)	10 (90.9%)	2 (100%)	12 (100%)
	Non-Endometrioid	1 (5.3%)	0	1 (9.1%)	0	0
Tumor Grade	1	18 (94.7%)	7 (70%)	7 (63.6%)	2 (100%)	11 (91.7%)
	2	1 (5.3%)	2 (20%)	1 (9.1%)	0	1 (8.3%)
	3	0	1 (10%)	3 (27.3%)	0	0
Mean SLNs Removed ± SD (SLNs Confirmed in Final Pathology)		5 ± 2	5.1 ± 1.8	4.9 ± 2.1	5 ± 1.5	5.3 ± 1.9
LVSI Present		1 (5.3%)	0	1 (9.1%)	0	1 (8.3%)
FIGO Stage 2023	IA1	2 (10.5%)	0	1 (9.05%)	1 (50%)	2 (16.7%)
	IA2	11 (57.9%)	7 (70%)	3 (27.3%)	1 (50%)	6 (50%)
	IB	3 (15.7%)	2 (20%)	3 (27.3%)	0	4 (33.3%)
	IIA	0	0	1 (9.05%)	0	0
	IIB	1 (5.3%)	1 (10%)	0	0	0
	IIIA2	1 (5.3%)	0	0	0	0
	IIIC1i	1 (5.3%)	0	3 (27.3%)	0	0
Recurrence during Follow-Up		0	0	0	0	0
Deaths, Cause (*n*, %)		1 (5.3%) myocardial infarction (non-cancer-related)	0	0	0	0

Abbreviations: BMI: body mass index, SLN: sentinel lymph node, SD: standard deviation, LVSI: lymphovascular space invasion, FIGO: International Federation of Gynecology and Obstetrics. Values are expressed as *n* (%) unless otherwise stated.

## Data Availability

The data presented in this study are available in this article and Supplementary Materials.

## Supplementary Materials

The following supporting information can be downloaded at: https://www.mdpi.com/article/10.3390/cancers17213570/s1, Supplementary File S1: Statistical analysis.

## Abbreviations

The following abbreviations are used in this manuscript:

EC	Endometrial cancer
USC	Uterine serous carcinoma
FIGO	International Federation of Gynecology and Obstetrics
BMI	Body mass index
EEC	Early-stage endometrial cancer
TH BSO	Total hysterectomy with bilateral salpingo-oophorectomy
SLNB	Sentinel lymph node biopsy
SLN	Sentinel lymph node
ICG	Indocyanine green
WHO	World Health Organization
ASA	American Society of Anesthesiology
LVSI	Lymphovascular space invasion
SD	Standard deviation
LOS	Length of stay
Km/m^2^	Kilograms per meter squared
