# Supplementary material for: Impact of Body Mass Index on Robotic Surgery Outcomes in Early-Stage Endometrial Cancer: A Retrospective Cohort Study
Source: Cancers (Basel). 2025 Nov 5;17(21):3570. doi: 10.3390/cancers17213570 (PMC12608680; doi:10.3390/cancers17213570)
Supplement: Supplementary file 1 [file cancers-17-03570-s001.zip › cancers-3910264-supplementary.pdf]

**Supplementary Material File S1: Statistical Analyses**

## 1. Descriptive Statistics

Descriptive statistics for Age, BMI, ASA classification, and Console Time (minutes) in the study cohort (n=54):

- Age: Mean = 59.69, SD = 11.28, Range = 38–84
- BMI: Mean = 31.09, SD = 9.42, Range = 19.10–53.68
- ASA Classification: Mean = 1.83, SD = 0.77, Range = 1–3
- Console Time (min): Mean = 121.37, SD = 46.68, Range = 52–250

**Descriptive Statistics**

| | N | Minimum | Maximum | Mean | Std. Deviation |
|---|---|---|---|---|---|
| Age | 54 | 38 | 84 | 59.69 | 11.282 |
| BMI | 54 | 19.10 | 53.68 | 31.0915 | 9.41825 |
| ASAClassification | 54 | 1 | 3 | 1.83 | .771 |
| Console Time(min) | 54 | 52 | 250 | 121.37 | 46.681 |
| Valid N (listwise) | 54 | | | | |

## 2. Console Time Analyses by BMI WHO Categories

### 2.1 Mean Console Time per BMI Category

- Normal (n=19): 125.11 ± 56.70 min
- Overweight (n=10): 110.20 ± 35.10 min
- Obese Class I (n=11): 127.00 ± 45.46 min

- Obese Class II (n=2): 131.00 ± 69.30 min
- Obese Class III (n=12): 118.00 ± 41.44 min
- Total (n=54): 121.37 ± 46.68 min

**Case Processing Summary**

| | | Cases | | | | | |
|---|---|---|---|---|---|---|---|
| | | Valid | | Missing | | Total | |
| | bmi_who | N | Percent | N | Percent | N | Percent |
| Console Time(min) | Normal | 19 | 100.0% | 0 | 0.0% | 19 | 100.0% |
| | Overweight | 10 | 100.0% | 0 | 0.0% | 10 | 100.0% |
| | Obese Class I | 11 | 100.0% | 0 | 0.0% | 11 | 100.0% |
| | Obese Class II | 2 | 100.0% | 0 | 0.0% | 2 | 100.0% |
| | Obese Class III | 12 | 100.0% | 0 | 0.0% | 12 | 100.0% |

**Case Processing Summary**

| | Cases | | | | | |
|---|---|---|---|---|---|---|
| | Included | | Excluded | | Total | |
| | N | Percent | N | Percent | N | Percent |
| Console Time(min) * bmi_who | 54 | 5.4% | 941 | 94.6% | 995 | 100.0% |

**Report**

Console Time(min)

| bmi_who | Mean | N | Std. Deviation |
|---|---|---|---|
| Normal | 125.11 | 19 | 56.696 |
| Overweight | 110.20 | 10 | 35.102 |
| Obese Class I | 127.00 | 11 | 45.464 |
| Obese Class | 131.00 | 2 | 69.296 |

| | | | |
|---|---|---|---|
| II | | | |
| Obese Class III | 118.00 | 12 | 41.438 |
| Total | 121.37 | 54 | 46.681 |

## 2.2 One-Way ANOVA Results

- $F(4,49) = 0.236$, $p = 0.917$ (not statistically significant)
- Effect sizes:
  - $Eta^2 = 0.019$
  - $Epsilon^2 = -0.061$
  - $Omega^2$ (Fixed) = -0.060

**Descriptives**

Console Time(min)

| | N | Mean | Std. Deviation | Std. Error | 95% Confidence Interval for Mean Lower Bound | 95% Confidence Interval for Mean Upper Bound | Minimum | Maximum |
|---|---|---|---|---|---|---|---|---|
| Normal | 19 | 125.11 | 56.696 | 13.007 | 97.78 | 152.43 | 52 | 250 |
| Overweight | 10 | 110.20 | 35.102 | 11.100 | 85.09 | 135.31 | 73 | 180 |
| Obese Class I | 11 | 127.00 | 45.464 | 13.708 | 96.46 | 157.54 | 70 | 196 |
| Obese Class II | 2 | 131.00 | 69.296 | 49.000 | -491.60 | 753.60 | 82 | 180 |
| Obese Class III | 12 | 118.00 | 41.438 | 11.962 | 91.67 | 144.33 | 59 | 200 |
| Total | 54 | 121.37 | 46.681 | 6.352 | 108.63 | 134.11 | 52 | 250 |

**ANOVA**

Console Time(min)

| | Sum of Squares | df | Mean Square | F | Sig. |
|---|---|---|---|---|---|
| Between Groups | 2183.203 | 4 | 545.801 | .236 | .917 |
| Within Groups | 113309.389 | 49 | 2312.437 | | |
| Total | 115492.593 | 53 | | | |

**ANOVA Effect Sizes**[a,b]

| Point Estimate | 95% Confidence Interval | |
|---|---|---|
| | Lower | Upper |
| .019 | .000 | .050 |
| -.061 | -.082 | -.027 |
| -.060 | -.080 | -.027 |
| -.014 | -.019 | -.007 |

a. Eta-squared and Epsilon-squared are estimated based on the fixed-effect model.

b. Negative but less biased estimates are retained, not rounded to zero.

## 3. Length of Stay (LOS) Analyses

### 3.1 Mean LOS per BMI Category

- Normal (n=19): 1.32 ± 0.58 days
- Overweight (n=10): 1.30 ± 0.48 days
- Obese Class I (n=11): 1.55 ± 0.93 days

- Obese Class II (n=2): 1.00 ± 0.00 days
- Obese Class III (n=12): 1.67 ± 1.23 days
- Total (n=54): 1.43 ± 0.82 days

**Descriptives**

LengthOfStay (d)

| | N | Mean | Std. Deviation | Std. Error | 95% Confidence Interval for Mean Lower Bound | Upper Bound | Minimum | Maximum |
|---|---|---|---|---|---|---|---|---|
| Normal | 19 | 1.32 | .582 | .134 | 1.04 | 1.60 | 1 | 3 |
| Overweight | 10 | 1.30 | .483 | .153 | .95 | 1.65 | 1 | 2 |
| Obese Class I | 11 | 1.55 | .934 | .282 | .92 | 2.17 | 1 | 4 |
| Obese Class II | 2 | 1.00 | .000 | .000 | 1.00 | 1.00 | 1 | 1 |
| Obese Class III | 12 | 1.67 | 1.231 | .355 | .88 | 2.45 | 1 | 5 |
| Total | 54 | 1.43 | .815 | .111 | 1.20 | 1.65 | 1 | 5 |

### 3.2 One-Way ANOVA Results

- $F(4,49) = 0.585$, $p = 0.675$ (not statistically significant)
- Effect sizes:
  - $Eta^2 = 0.046$
  - $Epsilon^2 = -0.032$
  - $Omega^2$ (Fixed) = -0.032

**ANOVA**

LengthOfStay (d)

| | Sum of Squares | df | Mean Square | F | Sig. |
|---|---|---|---|---|---|
| Between Groups | 1.605 | 4 | .401 | .585 | .675 |
| Within Groups | 33.599 | 49 | .686 | | |
| Total | 35.204 | 53 | | | |

**ANOVA Effect Sizes**[a,b]

| | | Point Estimate | 95% Confidence Interval | |
|---|---|---|---|---|
| | | | Lower | Upper |
| LengthOfStay (d) | Eta-squared | .046 | .000 | .124 |
| | Epsilon-squared | -.032 | -.082 | .052 |
| | Omega-squared Fixed-effect | -.032 | -.080 | .051 |
| | Omega-squared Random-effect | -.008 | -.019 | .013 |

a. Eta-squared and Epsilon-squared are estimated based on the fixed-effect model.

b. Negative but less biased estimates are retained, not rounded to zero.

## 4. Correlation Analysis

Pearson correlations between BMI, Console Time, SLNs Identified, and LOS:

- BMI & Console Time: r = .019, p = .890
- BMI & SLNs Identified: r = .076, p = .587

- BMI & LOS: r = .188, p = .174
- Console Time & SLNs Identified: r = .302, p = .026*
- Console Time & LOS: r = .261, p = .057
- SLNs Identified & LOS: r = .019, p = .892

* Statistically significant at $p < 0.05$

**Correlations**

| | | BMI | Console Time(min) | Total Number of SLNs Identified | LengthOfStay (d) |
|---|---|---|---|---|---|
| BMI | Pearson Correlation | 1 | .019 | .076 | .188 |
| | Sig. (2-tailed) | | .890 | .587 | .174 |
| | N | 54 | 54 | 54 | 54 |
| Console Time(min) | Pearson Correlation | .019 | 1 | .302* | .261 |
| | Sig. (2-tailed) | .890 | | .026 | .057 |
| | N | 54 | 54 | 54 | 54 |
| Total Number of SLNs Identified | Pearson Correlation | .076 | .302* | 1 | .019 |
| | Sig. (2-tailed) | .587 | .026 | | .892 |
| | N | 54 | 54 | 54 | 54 |
| LengthOfStay (d) | Pearson Correlation | .188 | .261 | .019 | 1 |
| | Sig. (2-tailed) | .174 | .057 | .892 | |
| | N | 54 | 54 | 54 | 54 |

*. Correlation is significant at the 0.05 level (2-tailed).

## 5. Console Time and Length of Stay (LOS) Groups by BMI Categories

### 5.1 Console Time Group (<120 vs ≥120 min)

Chi-square test evaluating the proportion of patients with short (<120 min) vs long (≥120 min) console times across BMI groups.

- $\chi^2(4) = 2.183$, $p = 0.702$
- No significant association found between BMI and console time category.

### 5.2 LOS Group (≤1 day vs >1 day)

Chi-square test evaluating the distribution of short (≤1 day) vs long (>1 day) hospital stay across BMI groups.

- $\chi^2(4) = 1.261$, $p = 0.868$
- No significant association found between BMI and LOS group.

## 6. Sentinel Lymph Node (SLN) Detection and Location by BMI Categories

### 6.1 SLN Detection (Bilateral / Unilateral / None)

- $\chi^2(8) = 6.208$, $p = 0.624$
- Majority had bilateral SLN detection across all BMI groups.
- No significant difference in SLN detection by BMI category.

**SLN Detection (bilateral/unilateral/none) * bmi_who**

**Crosstab**

| | | | bmi_who | | | |
|---|---|---|---|---|---|---|
| | | | Normal | Overweight | Obese Class I | Obese Class II |
| SLN Detection (bilateral/unilateral | Bilateral | Count | 17 | 9 | 9 | 1 |
| | | % within SLN | 36.2% | 19.1% | 19.1% | 2.1% |

| | | | | | | |
|---|---|---|---|---|---|---|
| /none) | | Detection (bilateral/unilateral /none) | | | | |
| | | % within bmi_who | 89.5% | 90.0% | 81.8% | 50.0% |
| | None | Count | 1 | 0 | 1 | 0 |
| | | % within SLN Detection (bilateral/unilateral /none) | 50.0% | 0.0% | 50.0% | 0.0% |
| | | % within bmi_who | 5.3% | 0.0% | 9.1% | 0.0% |
| | Unilateral | Count | 1 | 1 | 1 | 1 |
| | | % within SLN Detection (bilateral/unilateral /none) | 20.0% | 20.0% | 20.0% | 20.0% |
| | | % within bmi_who | 5.3% | 10.0% | 9.1% | 50.0% |
| Total | | Count | 19 | 10 | 11 | 2 |
| | | % within SLN Detection (bilateral/unilateral /none) | 35.2% | 18.5% | 20.4% | 3.7% |
| | | % within bmi_who | 100.0% | 100.0% | 100.0% | 100.0% |

**Crosstab**

| | | | bmi_who | |
|---|---|---|---|---|
| | | | Obese Class III | Total |
| SLN Detection (bilateral/unilateral/ none) | Bilateral | Count | 11 | 47 |
| | | % within SLN Detection (bilateral/unilateral/ | 23.4% | 100.0% |

| | | | | |
|---|---|---|---|---|
| | | none) | | |
| | | % within bmi_who | 91.7% | 87.0% |
| | None | Count | 0 | 2 |
| | | % within SLN Detection (bilateral/unilateral/ none) | 0.0% | 100.0% |
| | | % within bmi_who | 0.0% | 3.7% |
| | Unilateral | Count | 1 | 5 |
| | | % within SLN Detection (bilateral/unilateral/ none) | 20.0% | 100.0% |
| | | % within bmi_who | 8.3% | 9.3% |
| Total | | Count | 12 | 54 |
| | | % within SLN Detection (bilateral/unilateral/ none) | 22.2% | 100.0% |
| | | % within bmi_who | 100.0% | 100.0% |

**Chi-Square Tests**

| | Value | df | Asymptotic Significance (2-sided) |
|---|---|---|---|
| Pearson Chi-Square | 6.208[a] | 8 | .624 |
| Likelihood Ratio | 5.108 | 8 | .746 |
| N of Valid Cases | 54 | | |

a. 11 cells (73.3%) have expected count less than 5. The minimum expected count is .07.

### 6.2 Binary SLN Detection (Detected vs Not Found)

- $\chi^2(4) = 1.948$, $p = 0.745$
- Binary SLN detection (any vs none) was not significantly associated with BMI.

**Bilateral or Unilateral=1, Not Found=0 * bmi_who**

**Crosstab**

| | | | bmi_who | | |
|---|---|---|---|---|---|
| | | | Normal | Overweight | Obese Class I |
| Bilateral or Unilateral=1, Not Found=0 | None | Count | 1 | 0 | 1 |
| | | % within Bilateral or Unilateral=1, Not Found=0 | 50.0% | 0.0% | 50.0% |
| | | % within bmi_who | 5.3% | 0.0% | 9.1% |
| | Bilateral or Unilateral | Count | 18 | 10 | 10 |
| | | % within Bilateral or Unilateral=1, Not Found=0 | 34.6% | 19.2% | 19.2% |
| | | % within bmi_who | 94.7% | 100.0% | 90.9% |
| Total | | Count | 19 | 10 | 11 |
| | | % within Bilateral or Unilateral=1, Not Found=0 | 35.2% | 18.5% | 20.4% |
| | | % within bmi_who | 100.0% | 100.0% | 100.0% |

**Crosstab**

| | | | bmi_who | | |
|---|---|---|---|---|---|
| | | | Obese Class II | Obese Class III | Total |
| Bilateral or Unilateral=1, Not Found=0 | None | Count | 0 | 0 | 2 |
| | | % within Bilateral or Unilateral=1, Not Found=0 | 0.0% | 0.0% | 100.0% |
| | | % within bmi_who | 0.0% | 0.0% | 3.7% |
| | Bilateral or Unilateral | Count | 2 | 12 | 52 |
| | | % within Bilateral or Unilateral=1, Not Found=0 | 3.8% | 23.1% | 100.0% |
| | | % within bmi_who | 100.0% | 100.0% | 96.3% |
| Total | | Count | 2 | 12 | 54 |
| | | % within Bilateral or Unilateral=1, Not Found=0 | 3.7% | 22.2% | 100.0% |
| | | % within bmi_who | 100.0% | 100.0% | 100.0% |

**Chi-Square Tests**

| | Value | df | Asymptotic Significance (2-sided) |
|---|---|---|---|
| Pearson Chi-Square | 1.948[a] | 4 | .745 |
| Likelihood Ratio | 2.571 | 4 | .632 |
| Linear-by-Linear Association | .304 | 1 | .581 |

| | | | |
|---|---|---|---|
| N of Valid Cases | 54 | | |

a. 6 cells (60.0%) have expected count less than 5. The minimum expected count is .07.

### 6.3 SLN Location (Obturator / External Iliac / Pre-sacral)

- $\chi^2(8) = 8.821$, $p = 0.358$
- SLNs were most commonly found in the external iliac region across all BMI groups.
- No significant difference in anatomical distribution of SLNs by BMI.

**SLN Location (e.g., obturator, external iliac) * bmi_who**

**Crosstab**

| | | | bmi_who | | |
|---|---|---|---|---|---|
| | | | Normal | Overweight | Obese Class I |
| SLN Location (e.g., obturator, external iliac) | external iliac | Count | 11 | 8 | 8 |
| | | % within SLN Location (e.g., obturator, external iliac) | 29.7% | 21.6% | 21.6% |
| | | % within bmi_who | 57.9% | 80.0% | 72.7% |
| | obturator | Count | 7 | 2 | 3 |
| | | % within SLN Location (e.g., obturator, external iliac) | 43.8% | 12.5% | 18.8% |
| | | % within bmi_who | 36.8% | 20.0% | 27.3% |
| | pre-sacral | Count | 1 | 0 | 0 |

| | | | | | |
|---|---|---|---|---|---|
| | | % within SLN Location (e.g., obturator, external iliac) | 100.0% | 0.0% | 0.0% |
| | | % within bmi_who | 5.3% | 0.0% | 0.0% |
| Total | | Count | 19 | 10 | 11 |
| | | % within SLN Location (e.g., obturator, external iliac) | 35.2% | 18.5% | 20.4% |
| | | % within bmi_who | 100.0% | 100.0% | 100.0% |

**Crosstab**

| | | | bmi_who | | |
|---|---|---|---|---|---|
| | | | Obese Class II | Obese Class III | Total |
| SLN Location (e.g., obturator, external iliac) | external iliac | Count | 0 | 10 | 37 |
| | | % within SLN Location (e.g., obturator, external iliac) | 0.0% | 27.0% | 100.0% |
| | | % within bmi_who | 0.0% | 83.3% | 68.5% |
| | obturator | Count | 2 | 2 | 16 |
| | | % within SLN Location (e.g., obturator, external iliac) | 12.5% | 12.5% | 100.0% |
| | | % within bmi_who | 100.0% | 16.7% | 29.6% |
| | pre-sacral | Count | 0 | 0 | 1 |
| | | % within SLN Location (e.g., obturator, external | 0.0% | 0.0% | 100.0% |

| | | | | |
|---|---|---|---|---|
| | iliac) | | | |
| | % within bmi_who | 0.0% | 0.0% | 1.9% |
| Total | Count | 2 | 12 | 54 |
| | % within SLN Location (e.g., obturator, external iliac) | 3.7% | 22.2% | 100.0% |
| | % within bmi_who | 100.0% | 100.0% | 100.0% |

**Chi-Square Tests**

| | Value | df | Asymptotic Significance (2-sided) |
|---|---|---|---|
| Pearson Chi-Square | 8.821[a] | 8 | .358 |
| Likelihood Ratio | 9.275 | 8 | .320 |
| N of Valid Cases | 54 | | |

a. 10 cells (66.7%) have expected count less than 5. The minimum expected count is .04.

**7. Binary Logistic Regression for SLN Detection**

Binary logistic regression model predicting SLN detection (binary: detected vs not found) based on Age, BMI, ASA classification, previous abdominal surgeries, and tumor size.

Model Fit:
- Omnibus $\chi^2(5) = 17.108$, p = 0.004
- Nagelkerke $R^2 = 1.000$ (perfect prediction, but likely due to small sample size or convergence issue)

Block 1: Regression Coefficients:

- Age: B = -9.691, p = 0.995
- BMI: B = 14.469, p = 0.998
- ASA: B = -26.680, p = 0.999
- Previous surgeries: B = -66.370, p = 0.995
- Tumor size: B = -11.683, p = 1.000
- Constant: B = 466.132, p = 0.998

⚠️ Note: Model did not converge properly (iteration limit reached). Interpret results with caution.

**Case Processing Summary**

| Unweighted Cases[a] | | N | Percent |
|---|---|---|---|
| Selected Cases | Included in Analysis | 54 | 5.4 |
| | Missing Cases | 941 | 94.6 |
| | Total | 995 | 100.0 |
| Unselected Cases | | 0 | .0 |
| Total | | 995 | 100.0 |

a. If weight is in effect, see classification table for the total number of cases.

**Dependent Variable Encoding**

| Original Value | Internal Value |
|---|---|
| .00 | 0 |
| 1.00 | 1 |

**Block 0: Beginning Block**

**Classification Table**[a,b]

| Observed | | | Predicted: Bilateral or Unilateral=1, Not Found=0 .00 | Predicted: Bilateral or Unilateral=1, Not Found=0 1.00 | Percentage Correct |
|---|---|---|---|---|---|
| Step 0 | Bilateral or Unilateral=1, Not Found=0 | .00 | 0 | 2 | .0 |
| | | 1.00 | 0 | 52 | 100.0 |
| | Overall Percentage | | | | 96.3 |

a. Constant is included in the model.

b. The cut value is .500

**Variables in the Equation**

| | | B | S.E. | Wald | df | Sig. | Exp(B) |
|---|---|---|---|---|---|---|---|
| Step 0 | Constant | 3.258 | .721 | 20.444 | 1 | <.001 | 26.000 |

**Variables not in the Equation**

| | | | Score | df | Sig. |
|---|---|---|---|---|---|
| Step 0 | Variables | Age | 6.203 | 1 | .013 |
| | | BMI | .283 | 1 | .595 |
| | | ASAClassification | .099 | 1 | .753 |
| | | Number of Previous Abdomnal Surgeries (Total) | .931 | 1 | .335 |
| | | Tumor Size(cm) | .275 | 1 | .600 |
| | Overall Statistics | | 8.436 | 5 | .134 |

**Block 1: Method = Enter**

**Omnibus Tests of Model Coefficients**

| | | Chi-square | df | Sig. |
|---|---|---|---|---|
| Step 1 | Step | 17.108 | 5 | .004 |
| | Block | 17.108 | 5 | .004 |
| | Model | 17.108 | 5 | .004 |

**Model Summary**

| Step | -2 Log likelihood | Cox & Snell R Square | Nagelkerke R Square |
|---|---|---|---|
| 1 | .000[a] | .272 | 1.000 |

a. Estimation terminated at iteration number 20 because maximum iterations has been reached. Final solution cannot be found.

**Classification Table[a]**

| | | | Predicted | | |
|---|---|---|---|---|---|
| | | | Bilateral or Unilateral=1, Not Found=0 | | Percentage Correct |
| | Observed | | .00 | 1.00 | |
| Step 1 | Bilateral or Unilateral=1, Not Found=0 | .00 | 2 | 0 | 100.0 |
| | | 1.00 | 0 | 52 | 100.0 |
| | Overall Percentage | | | | 100.0 |

a. The cut value is .500

**Variables in the Equation**

| | | B | S.E. | Wald | df | Sig. | Exp(B) |
|---|---|---|---|---|---|---|---|
| Step 1[a] | Age | -9.691 | 1617.576 | .000 | 1 | .995 | .000 |
| | BMI | 14.469 | 5887.632 | .000 | 1 | .998 | 1922022.718 |
| | ASAClassification | -26.680 | 14870.104 | .000 | 1 | .999 | .000 |
| | Number of Previous Abdomnal Surgeries (Total) | -66.370 | 10929.036 | .000 | 1 | .995 | .000 |
| | Tumor Size(cm) | -11.683 | 20802.123 | .000 | 1 | 1.000 | .000 |
| | Constant | 466.132 | 194054.268 | .000 | 1 | .998 | 2.744E+202 |

**Variables in the Equation**

| | | 95% C.I.for EXP(B) | |
|---|---|---|---|
| | | Lower | Upper |
| Step 1[a] | Age | .000 | . |
| | BMI | .000 | . |
| | ASAClassification | .000 | . |
| | Number of Previous Abdomnal Surgeries (Total) | .000 | . |
| | Tumor Size(cm) | .000 | . |
| | Constant | | |

a. Variable(s) entered on step 1: Age, BMI, ASAClassification, Number of Previous Abdomnal Surgeries (Total), Tumor Size(cm).

**8. Mean Console time between Morbidly Obese and Non- Morbidly Obese patients**

**Report**

Console Time(min)

| Obese | N | Mean | Median | Std. Deviation | Minimum | Maximum |
|---|---|---|---|---|---|---|
| Non-Morbidly Obese | 42 | 122.33 | 110.50 | 48.497 | 52 | 250 |
| Morbidly Obese | 12 | 118.00 | 113.00 | 41.438 | 59 | 200 |
| Total | 54 | 121.37 | 113.00 | 46.681 | 52 | 250 |

**9. Inferential test (Independent Samples t-test): Comparing mean Console time between Morbidly Obese and Non- Morbidly Obese patients**

**Case Processing Summary**

| | Cases | | | | | |
|---|---|---|---|---|---|---|
| | Included | | Excluded | | Total | |
| | N | Percent | N | Percent | N | Percent |
| Console Time(min) * Obese | 54 | 0.0% | 1048429 | 100.0% | 1048483 | 100.0% |

**Report**

Console Time(min)

| Obese | N | Mean | Median | Std. Deviation | Minimum | Maximum |
|---|---|---|---|---|---|---|
| Non-Severe Obesity | 42 | 122.33 | 110.50 | 48.497 | 52 | 250 |
| Severe Obesity | 12 | 118.00 | 113.00 | 41.438 | 59 | 200 |
| Total | 54 | 121.37 | 113.00 | 46.681 | 52 | 250 |

**T-Test**

**Group Statistics**

| | Severe Obese | N | Mean | Std. Deviation | Std. Error Mean |
|---|---|---|---|---|---|
| Console Time(min) | No | 42 | 122.33 | 48.497 | 7.483 |
| | Yes | 12 | 118.00 | 41.438 | 11.962 |

**Independent Samples Test**

| | | Levene's Test for Equality of Variances | | t-test for Equality of Means | | | | | | | |
|---|---|---|---|---|---|---|---|---|---|---|---|
| | | | | | | Significance | | | | 95% Confidence Interval of the Difference | |
| | | F | Sig. | t | df | One-Sided p | Two-Sided p | Mean Difference | Std. Error Difference | Lower | Upper |
| Console Time( | Equal variances assumed | 1.349 | .251 | .281 | 52 | .390 | .780 | 4.333 | 15.414 | -26.598 | 35.265 |

| | | | | | | | | | | |
|---|---|---|---|---|---|---|---|---|---|---|
| min) | Equal variances not assumed | | | .307 | 20.454 | .381 | .762 | 4.333 | 14.110 | -25.058 | 33.724 |

**Independent Samples Effect Sizes**

| | | Standardizer[a] | Point Estimate | 95% Confidence Interval Lower | 95% Confidence Interval Upper |
|---|---|---|---|---|---|
| Console Time(min) | Cohen's d | 47.092 | .092 | -.550 | .733 |
| | Hedges' correction | 47.785 | .091 | -.542 | .723 |
| | Glass's delta | 41.438 | .105 | -.541 | .745 |

a. The denominator used in estimating the effect sizes.
Cohen's d uses the pooled standard deviation.
Hedges' correction uses the pooled standard deviation, plus a correction factor.
Glass's delta uses the sample standard deviation of the control group.

## 10. Total Surgery Duration means between Morbidly Obese and Non- Morbidly Obese patients & between BMI WHO categories

**Case Processing Summary**

| | Cases Included N | Cases Included Percent | Cases Excluded N | Cases Excluded Percent | Cases Total N | Cases Total Percent |
|---|---|---|---|---|---|---|
| Total Case Duration (min) * BMI WHO | 54 | 0.0% | 1048429 | 100.0% | 1048483 | 100.0% |
| Total Case Duration (min) * Obese | 54 | 0.0% | 1048429 | 100.0% | 1048483 | 100.0% |

**Total Case Duration (min) * BMI WHO**

Total Case Duration (min)

| BMI WHO | N | Mean | Median | Std. Deviation | Minimum | Maximum |
|---|---|---|---|---|---|---|
| Normal | 19 | 192.7605 | 173.8000 | 55.58157 | 118.88 | 323.60 |
| Obese Class I | 11 | 195.8636 | 197.3000 | 48.18220 | 128.02 | 279.12 |
| Obese Class II | 2 | 199.1333 | 199.1333 | 61.89541 | 155.37 | 242.90 |
| Obese Class III | 12 | 184.5889 | 176.6333 | 41.81171 | 117.08 | 270.72 |
| Overweight | 10 | 176.3800 | 170.0917 | 34.46327 | 139.70 | 250.02 |
| Total | 54 | 188.7793 | 175.3250 | 46.66006 | 117.08 | 323.60 |

**Total Case Duration (min) * Obese**

Total Case Duration (min)

| Obese | N | Mean | Median | Std. Deviation | Minimum | Maximum |
|---|---|---|---|---|---|---|
| Non-Severe Obesity | 42 | 189.9766 | 175.3250 | 48.36048 | 118.88 | 323.60 |
| Severe Obesity | 12 | 184.5889 | 176.6333 | 41.81171 | 117.08 | 270.72 |
| Total | 54 | 188.7793 | 175.3250 | 46.66006 | 117.08 | 323.60 |

## 11. Inferential test (Independent Samples t-test): Comparing mean Total Surgery Duration between Morbidly Obese and Non-Morbidly Obese patients

**T-Test**

**Group Statistics**

| | Severe Obese | N | Mean | Std. Deviation | Std. Error Mean |
|---|---|---|---|---|---|
| Total Case Duration (min) | No | 42 | 189.9766 | 48.36048 | 7.46218 |
| | Yes | 12 | 184.5889 | 41.81171 | 12.07000 |

**Independent Samples Test**

| | | Levene's Test for Equality of Variances | | t-test for Equality of Means | | | | | | | |
|---|---|---|---|---|---|---|---|---|---|---|---|
| | | | | | | Significance | | | | 95% Confidence Interval of the Difference | |
| | | F | Sig. | t | df | One-Sided p | Two-Sided p | Mean Difference | Std. Error Difference | Lower | Upper |
| Total Case Duration | Equal variances assumed | .882 | .352 | .350 | 52 | .364 | .728 | 5.38770 | 15.40113 | -25.51694 | 36.29233 |

| | | | | | | | | | | | |
|---|---|---|---|---|---|---|---|---|---|---|---|
| (min) | Equal variances not assumed | | | .380 | 20.223 | .354 | .708 | 5.38770 | 14.19046 | -24.19214 | 34.96754 |

**Independent Samples Effect Sizes**

| | | Standardizer[a] | Point Estimate | 95% Confidence Interval | |
|---|---|---|---|---|---|
| | | | | Lower | Upper |
| Total Case Duration (min) | Cohen's d | 47.05124 | .115 | -.528 | .756 |
| | Hedges' correction | 47.74374 | .113 | -.520 | .745 |
| | Glass's delta | 41.81171 | .129 | -.518 | .770 |

a. The denominator used in estimating the effect sizes.

Cohen's d uses the pooled standard deviation.

Hedges' correction uses the pooled standard deviation, plus a correction factor.

Glass's delta uses the sample standard deviation of the control group.

## 12. Non Console Time means between Morbidly Obese and Non- Morbidly Obese patients & between BMI WHO categories

### Means

**Case Processing Summary**

| | Cases | | | | | |
|---|---|---|---|---|---|---|
| | Included | | Excluded | | Total | |
| | N | Percent | N | Percent | N | Percent |
| NonConsoleTime * BMI WHO | 54 | 0.0% | 1048429 | 100.0% | 1048483 | 100.0% |
| NonConsoleTime * Obese | 54 | 0.0% | 1048429 | 100.0% | 1048483 | 100.0% |

**NonConsoleTime * BMI WHO**

NonConsoleTime

| BMI WHO | N | Mean | Median | Std. Deviation | Minimum | Maximum |
|---|---|---|---|---|---|---|
| Normal | 19 | 67.6558 | 67.8000 | 7.70799 | 55.55 | 80.12 |
| Obese Class I | 11 | 68.8645 | 66.5500 | 9.32655 | 55.55 | 83.12 |
| Obese Class II | 2 | 68.1350 | 68.1350 | 7.40341 | 62.90 | 73.37 |
| Obese Class III | 12 | 66.5900 | 66.4650 | 8.48649 | 54.92 | 80.12 |
| Overweight | 10 | 66.1810 | 66.9100 | 8.09614 | 49.07 | 79.08 |
| Total | 54 | 67.4098 | 67.0000 | 8.03869 | 49.07 | 83.12 |

**NonConsoleTime * Obese**

NonConsoleTime

| Obese | N | Mean | Median | Std. Deviation | Minimum | Maximum |
|---|---|---|---|---|---|---|
| Non-Severe Obesity | 42 | 67.6440 | 67.0000 | 7.99740 | 49.07 | 83.12 |
| Severe Obesity | 12 | 66.5900 | 66.4650 | 8.48649 | 54.92 | 80.12 |
| Total | 54 | 67.4098 | 67.0000 | 8.03869 | 49.07 | 83.12 |

**13. Inferential test (Independent Samples t-test): Comparing mean Console time between Morbidly Obese and Non- Morbidly Obese patients**

**T-Test**

**Group Statistics**

| | Severe Obese | N | Mean | Std. Deviation | Std. Error Mean |
|---|---|---|---|---|---|
| NonConsoleTime | No | 42 | 67.6440 | 7.99740 | 1.23403 |
| | Yes | 12 | 66.5900 | 8.48649 | 2.44984 |

**Independent Samples Test**

| | Levene's Test for Equality of Variances | | t-test for Equality of Means | | | | | |
|---|---|---|---|---|---|---|---|---|
| | F | Sig. | t | df | Significance | Mean Difference | Std. Error Difference | 95% Confidence Interval of the Difference |

| | | | | | | One-Sided p | Two-Sided p | | | Lower | Upper |
|---|---|---|---|---|---|---|---|---|---|---|---|
| NonConsoleTime | Equal variances assumed | .144 | .706 | .397 | 52 | .346 | .693 | 1.05405 | 2.65244 | -4.26845 | 6.37655 |
| | Equal variances not assumed | | | .384 | 16.997 | .353 | .706 | 1.05405 | 2.74309 | -4.73345 | 6.84154 |

**Independent Samples Effect Sizes**

| | | Standardizer[a] | Point Estimate | 95% Confidence Interval | |
|---|---|---|---|---|---|
| | | | | Lower | Upper |
| NonConsoleTime | Cohen's d | 8.10332 | .130 | -.513 | .771 |
| | Hedges' correction | 8.22259 | .128 | -.505 | .760 |
| | Glass's delta | 8.48649 | .124 | -.522 | .765 |

a. The denominator used in estimating the effect sizes.

Cohen's d uses the pooled standard deviation.

Hedges' correction uses the pooled standard deviation, plus a correction factor.

Glass's delta uses the sample standard deviation of the control group.

## 14. Length of Stay means between Morbidly Obese and Non-Morbidly Obese patients

### Means

**Case Processing Summary**

| | Cases | | | | | |
|---|---|---|---|---|---|---|
| | Included | | Excluded | | Total | |
| | N | Percent | N | Percent | N | Percent |
| LengthOfStay (d) * Obese | 54 | 0.0% | 1048429 | 100.0% | 1048483 | 100.0% |

**Report**

LengthOfStay (d)

| Obese | N | Mean | Median | Std. Deviation | Minimum | Maximum |
|---|---|---|---|---|---|---|
| Non-Severe Obesity | 42 | 1.36 | 1.00 | .656 | 1 | 4 |
| Severe Obesity | 12 | 1.67 | 1.00 | 1.231 | 1 | 5 |
| Total | 54 | 1.43 | 1.00 | .815 | 1 | 5 |

**15. Inferential test (Independent Samples t-test): Comparing mean Length of Stay between Morbidly Obese and Non- Morbidly Obese patients**

**T-Test**

**Group Statistics**

| | Severe Obese | N | Mean | Std. Deviation | Std. Error Mean |
|---|---|---|---|---|---|
| LengthOfStay (d) | No | 42 | 1.36 | .656 | .101 |
| | Yes | 12 | 1.67 | 1.231 | .355 |

**Independent Samples Test**

| | | Levene's Test for Equality of Variances | | t-test for Equality of Means | | | | | | | |
|---|---|---|---|---|---|---|---|---|---|---|---|
| | | | | | | Significance | | | | 95% Confidence Interval of the Difference | |
| | | F | Sig. | t | df | One-Sided p | Two-Sided p | Mean Difference | Std. Error Difference | Lower | Upper |
| LengthOfStay (d) | Equal variances assumed | 5.009 | .030 | -1.164 | 52 | .125 | .250 | -.310 | .266 | -.843 | .224 |
| | Equal variances not assumed | | | -.838 | 12.835 | .209 | .417 | -.310 | .369 | -1.109 | .490 |

**Independent Samples Effect Sizes**

| | | Standardizer[a] | Point Estimate | 95% Confidence Interval | |
|---|---|---|---|---|---|
| | | | | Lower | Upper |
| LengthOfStay (d) | Cohen's d | .812 | -.381 | -1.025 | .266 |
| | Hedges' correction | .824 | -.376 | -1.010 | .263 |
| | Glass's delta | 1.231 | -.251 | -.896 | .404 |

a. The denominator used in estimating the effect sizes.

Cohen's d uses the pooled standard deviation.

Hedges' correction uses the pooled standard deviation, plus a correction factor.

Glass's delta uses the sample standard deviation of the control group.

## 16. Correlation Matrix Analysis

### Correlations

**Correlations**

| | | BMI | Console Time(min) | NonConsoleTime | Total Case Duration (min) | Total Number of SLNs Identified | LengthOfStay (d) |
|---|---|---|---|---|---|---|---|
| BMI | Pearson Correlation | 1 | .019 | -.035 | .013 | .076 | .188 |
| | Sig. (2-tailed) | | .890 | .804 | .924 | .587 | .174 |
| | N | 54 | 54 | 54 | 54 | 54 | 54 |
| Console Time(min) | Pearson Correlation | .019 | 1 | -.089 | .985** | .302* | .261 |
| | Sig. (2-tailed) | .890 | | .524 | <.001 | .026 | .057 |

| | | | | | | | |
|---|---|---|---|---|---|---|---|
| | N | 54 | 54 | 54 | 54 | 54 | 54 |
| NonConsoleTime | Pearson Correlation | -.035 | -.089 | 1 | .084 | -.003 | -.250 |
| | Sig. (2-tailed) | .804 | .524 | | .548 | .980 | .069 |
| | N | 54 | 54 | 54 | 54 | 54 | 54 |
| Total Case Duration (min) | Pearson Correlation | .013 | .985** | .084 | 1 | .302* | .218 |
| | Sig. (2-tailed) | .924 | <.001 | .548 | | .027 | .113 |
| | N | 54 | 54 | 54 | 54 | 54 | 54 |
| Total Number of SLNs Identified | Pearson Correlation | .076 | .302* | -.003 | .302* | 1 | .019 |
| | Sig. (2-tailed) | .587 | .026 | .980 | .027 | | .892 |
| | N | 54 | 54 | 54 | 54 | 54 | 54 |
| LengthOfStay (d) | Pearson Correlation | .188 | .261 | -.250 | .218 | .019 | 1 |
| | Sig. (2-tailed) | .174 | .057 | .069 | .113 | .892 | |
| | N | 54 | 54 | 54 | 54 | 54 | 54 |

**. Correlation is significant at the 0.01 level (2-tailed).

*. Correlation is significant at the 0.05 level (2-tailed).

**17. Total SLNs Identified means between Morbidly Obese and Non-Morbidly Obese patients & between BMI WHO categories**

**Means**

**Case Processing Summary**

| | Cases | | | | | |
|---|---|---|---|---|---|---|
| | Included | | Excluded | | Total | |
| | N | Percent | N | Percent | N | Percent |
| Total Number of SLNs Identified * Obese | 54 | 0.0% | 1048429 | 100.0% | 1048483 | 100.0% |

**Report**

Total Number of SLNs Identified

| Obese | N | Mean | Median | Std. Deviation | Minimum | Maximum |
|---|---|---|---|---|---|---|
| Non-Severe Obesity | 42 | 5.00 | 3.00 | 4.601 | 0 | 20 |
| Severe Obesity | 12 | 5.25 | 3.50 | 4.575 | 1 | 16 |
| Total | 54 | 5.06 | 3.00 | 4.553 | 0 | 20 |

## 18. Inferential test (Independent Samples t-test): Comparing mean Total SLNs Identified between Morbidly Obese and Non- Morbidly Obese patients

### T-Test

**Group Statistics**

| | Severe Obese | N | Mean | Std. Deviation | Std. Error Mean |
|---|---|---|---|---|---|
| Total Number of SLNs Identified | No | 42 | 5.00 | 4.601 | .710 |
| | Yes | 12 | 5.25 | 4.575 | 1.321 |

**Independent Samples Test**

| | Levene's Test for Equality of Variances | t-test for Equality of Means |
|---|---|---|

| | | F | Sig. | t | df | Significance | | Mean Difference | Std. Error Difference | 95% Confidence Interval of the Difference | |
|---|---|---|---|---|---|---|---|---|---|---|---|
| | | | | | | One-Sided p | Two-Sided p | | | Lower | Upper |
| Total Number of SLNs Identified | Equal variances assumed | .001 | .980 | -.166 | 52 | .434 | .869 | -.250 | 1.504 | -3.269 | 2.769 |
| | Equal variances not assumed | | | -.167 | 17.876 | .435 | .869 | -.250 | 1.499 | -3.402 | 2.902 |

**Independent Samples Effect Sizes**

| | | Standardizer[a] | Point Estimate | 95% Confidence Interval | |
|---|---|---|---|---|---|
| | | | | Lower | Upper |
| Total Number of SLNs Identified | Cohen's d | 4.596 | -.054 | -.696 | .587 |
| | Hedges' correction | 4.663 | -.054 | -.686 | .579 |
| | Glass's delta | 4.575 | -.055 | -.695 | .589 |

a. The denominator used in estimating the effect sizes.

Cohen's d uses the pooled standard deviation.

Hedges' correction uses the pooled standard deviation, plus a correction factor.

Glass's delta uses the sample standard deviation of the control group.
